# Understanding the experiences, perspectives and values of indigenous women around smoking cessation in pregnancy: systematic review and thematic synthesis of qualitative studies

**DOI:** 10.1186/s12939-019-0981-7

**Published:** 2019-05-22

**Authors:** Rachael C. Walker, Aria Graham, Suetonia C. Palmer, Anita Jagroop, David C. Tipene-Leach

**Affiliations:** 1 Eastern Institute of Technology, 501 Gloucester Street, Taradale, Napier, Hawke’s Bay, 4112 New Zealand; 2 Whakauae Research for Maori Health and Development, Whanganui, 4541 New Zealand; 3 Department of Medicine, University of Otago Christchurch, Christchurch, 8140 New Zealand

**Keywords:** Indigenous, Smoking, Pregnancy, Smoking cessation, Qualitative, Systematic review

## Abstract

**Background:**

The prevalence of smoking during pregnancy among indigenous women approaches 50% and is associated with sudden infant death, pregnancy loss, preterm delivery, low birth weight, and anatomical deformity. This study aims to synthesise qualitative studies by reporting experiences, perceptions, and values of smoking cessation among pregnant indigenous women to inform potential interventions.

**Method:**

A highly-sensitive search of MEDLINE, Embase, PsychINFO, and CINAHL, in conjunction with analysis of Google Scholar and reference lists of related studies was conducted in March 2018. We utilised two methods (thematic synthesis and an indigenous Māori analytical framework) in parallel to analyse data. Completeness of reporting in studies was evaluated using the Consolidated Criteria for Reporting Qualitative Studies (COREQ) framework.

**Results:**

We included seven studies from Australia and New Zealand involving 250 indigenous women. Three themes were identified. *Realising well-being and creating agency* included giving the best start to baby, pride in being a healthy mum, female role models, and family support. *Understanding the drivers for smoking* included the impact of stress and chaos that hindered prioritisation of self-care, the social acceptability of smoking, guilt and feeling judged, and inadequate information about the risks of smoking. Indigenous women strongly preferred *culturally responsive approaches* to smoking cessation, placing value on programs designed specifically for and by indigenous people, that were accessible, and provided an alternative to smoking.

**Conclusion:**

Future interventions and smoking cessation programmes might be more effective and acceptable to indigenous women and families when they harness self-agency and the desire for a healthy baby, recognise the high value of indigenous peer involvement, and embed a social focus in place of smoking as a way to maintain community support and relationships. Development and evaluation of smoking cessation programs for pregnant indigenous women and families is warranted.

## Background

About one in eight non-indigenous women smoke during pregnancy, while almost half of indigenous women smoke while pregnant [1–4]. Smoking in pregnancy increases pregnancy and neonatal complications including miscarriage, pre-term delivery, placental complications, low birth weight, and anatomical abnormalities [5, 6]. Smoking during pregnancy is a major risk factor for sudden unexpected death in infancy (SUDI), particularly when associated with infant bed sharing [7, 8] and this is well noted among indigenous babies [8–11].

Many smoking cessation initiatives have been developed to decrease smoking in pregnancy [12–16], including interventions specifically for indigenous women [14, 17]. Despite these interventions and related policies, programmes, and regulations, smoking among indigenous women remains a serious health problem internationally. Effective health interventions require an understanding of the social context of the problem and of the target audience. Understanding therefore, the experiences and values of indigenous women is crucial to ensuring that future interventions and policy better align with the preferences of indigenous women for smoking cessation. To date, many women have reported wanting to stop smoking but feeling unable to quit, being unaware of services or finding them difficult to access, and expressing concerns about being stigmatised once identified as a smoker [18–20].

This systematic review of qualitative studies aims to describe indigenous women’s experiences, perceptions, and values related to stopping smoking in pregnancy in order to inform the development of programs and interventions that respond to the needs, concerns and preferences of indigenous women.

## Methods

### Searches

Highly-sensitive searches were conducted in MEDLINE, Embase, PsychINFO, and CINAHL, from January 1, 2000 to March 1, 2018 Google Scholar and reference lists of related studies and reviews were also searched. Two authors (RCW, AJ) screened initial search results of titles and abstracts independently and excluded those that did not meet the review eligibility criteria. Full texts of potentially relevant articles were then screened by all authors.

### Study inclusion and exclusion criteria

Qualitative studies were eligible if they reported qualitative data about smoking cessation in pregnancy through interviews, focus groups, or other responses (e.g. diaries, observation) involving indigenous women. We included studies involving indigenous women from New Zealand, Australia, Canada, Taiwan, and the United States. Studies were ineligible if they solely included women who were not pregnant, not identified as indigenous, or did not address smoking cessation. Studies reporting structured questionnaires or surveys as the sole method for data collection or quantitative data were ineligible. Studies that did not elicit data directly from indigenous women as consumers were also excluded.

### Study quality assessment

This thematic synthesis is reported according to the Enhancing transparency in reporting the synthesis of qualitative research (ENTREQ) statement [21]. Thematic synthesis aims to provide an over-arching conceptual understanding of a topic through the systematic examination of the content and overlap provided in available qualitative studies. Completeness of methodological reporting was assessed independently by two researchers (RCW and AG) using the consolidated criteria for reporting qualitative research (COREQ) checklist [22]. The COREQ checklist includes domains to evaluate reporting of the research team and reflexivity, study design, and data analysis and reporting, to provide an assessment of the transferability of the primary study to a specific research setting.

### Data synthesis and presentation

We used two methods of data analysis in parallel. The first author used thematic synthesis as described by Thomas and Harden [23]. The second author applied an indigenous Māori analytical framework to examine the findings, employing a series of lenses derived from distinctive cultural epistemologies [24] framed around an indigenous model called Te Whetu that is based on five interconnected aspects (mind, body, spirit, family and land) [25]. This thematic analysis then included indigenous interpretations and description of findings, ensuring that they were robust from a cultural perspective.

Participant quotations and text under the “Results/Findings” or “Conclusion/Discussion” section of each study was imported into HyperRESEARCH (ResearchWare, INC 2009, version 3.0.3) software. Two authors (RCW and AG) then independently performed line-by-line coding of the findings of the primary studies, conceptualized the data, and inductively identified concepts using the two different frameworks. Concepts were then together grouped into agreed themes with subthemes. The second stage was to identify conceptual links among subthemes and themes (by RCW and AG) using a mind mapping approach to extend the findings offered by the primary studies and to develop an analytical thematic schema. Researcher triangulation was used, in which all authors independently reviewed the preliminary themes and analytical frameworks and discussed the addition or revision of themes amongst the research team.

## Results

**Fig. 1:**
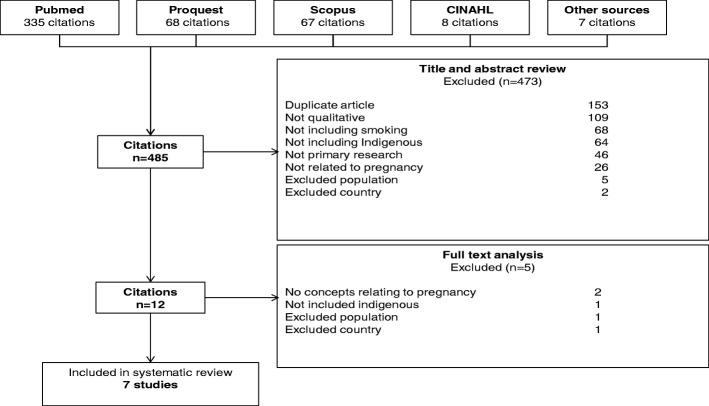
Search strategy and results of experiences, perspectives and values of indigenous women around smoking cessation in pregnancy

The search identified 485 unique records (Fig. 1). Of these, seven studies involving 250 pregnant indigenous women were included (Fig. 1). Four studies (57%) were from Australia, and three (two reporting from the same study) were conducted in New Zealand (43%). The participants’ age ranged from 14 to 53 years. Four studies involved face to face interviews, two used focus groups, and one study involved both methodologies (Table 1).

**Table 1:** Characteristics of Included Studies

Study	Country	Number of indigenous women participants (n)	Age range (years)	Methodological framework	Data collection	Analysis e.g. content, framework, thematic, GT	Topic
Bovill [29]	Australia	20	17–38	Therapeutic yarning	Face to face interviews	Inductive thematic analysis	Barriers to accepting smoking cessation support
Glover [30]^a^	New Zealand	60	17–43	“Te Whare Tapa Wha” – Indigenous theoretical framework	Semi-structured face to face interviews	Thematic analysis	Why Maori women continue to smoke during pregnancy
Glover [28]^a^	New Zealand	60	17–43	Not stated	Semi-structured face to face interviews	Thematic analysis	Perceptions of smoking cessations support
Glover [39]	New Zealand	92 (Maori) not specific	NS	Exploratory	Focus groups	General inductive approach	Motivation to quit
Gould, 2013 [26]	Australia	18 (15 women 3 men)	17–53	Not specified	Focus groups	Constant comparative method	Smoking in pregnancy and house
Gould, 2017 [27]	Australia	20	17–38	Yarning or conversational talking	Face to face interviews	Inductive	Narratives from smoking initiation through to pregnancy
Wood, 2008 [31]	Australia	40	14–50	Not specified	Focus groups and interviews	Thematic	Knowledge, cultural contexts and barriers to smoking cessation in pregnancy

NB: ^a^Same study. *NS* not specified

The comprehensiveness of reporting varied across the included studies (Table 2). The relationship established between the researchers and participants, member checking and whether repeat interviews were conducted was not described in any studies. Methods for recruiting women, the sample size, and participant quotations were reported in all seven studies. Two studies reported the presence of data saturation, field notes, and the duration of interviews.

**Table 2:** COREQ Assessment

Item	Studies reporting each item	Number of studies (%)
Personal Characteristics		
Interviewer / facilitator identified	[26, 27, 29, 39]	4 (57)
Occupation of the interview of facilitator	[26, 27, 29]	3 (43)
Experience or training in qualitative research	[27, 29]	2 (29)
Relationship with participants		
Relationship established prior to study commencement		0 (0)
Participant Selection		
Selection strategy *(*e.g. *snowball, purposive, convenience, comprehensive)*	[26–30, 39]	6 (86)
Method of approach or recruitment	[26–31, 39]	7 (100)
Sample size	[26–31, 39]	7 (100)
Number and/or reasons for non-participation	[27]	1 (14)
Setting		
Venue of data collection	[26, 27, 29, 31, 39]	5 (71)
Presence of non-participants *(*e.g. *clinical staff)*	[26, 29]	2 (29)
Description of the sample	[26–30, 39]	6 (86)
Data Collection		
Questions, prompts or topic guide	[26–31, 39]	7 (100)
Repeat interviews / observations		0 (0)
Audio / visual recording	[26, 27, 29, 39]	4 (57)
Field notes	[29, 39]	2 (29)
Duration of data collection (interview of focus group)	[29, 30]	2 (29)
Protocol for data preparation and transcription	[26, 27, 29, 39]	4 (57)
Data (or theoretical) saturation	[26, 27]	2 (29)
Data Analysis		
Researcher/expert triangulation (multiple researchers involved in coding and analysis)	[26, 27, 29, 39]	4 (57)
Derivation of themes or findings *(*e.g. *inductive, constant comparison)*	[26–29, 31, 39]	6 (86)
Use of software *(*e.g. *NVivo, HyperRESEARCH, Atlas.ti)*	[27, 29, 30]	3 (43)
Member checking (participant feedback on findings)	[27, 29]	2 (29)
Reporting		
Participant quotations or raw data provided *(picture, diary entries)*	[26–31, 39]	7 (100)
Range and depth of insight into participant perspectives *(thick description provided)*	[26–29, 31]	5 (71)

### Synthesis

Three themes were identified as central to indigenous women’s perspectives of smoking in pregnancy: realising well-being and creating agency, understanding the drivers for smoking, and appreciating culturally responsive approaches. The subthemes are described below, and we have indicated where possible, if findings were specific to a particular population or setting. Table 3 includes a selection of participant quotations and explanations provided by the authors to illustrate each theme.

**Table 3:** Patient Quotations of Experiences of

Themes/subthemes	Quotations
Realising well-being and creating agency	
Giving the best start to baby	“oh I’m pregnant I have to quit smoking” [26]
	“I would, I would quit straight away if I found out I was pregnant, cause that would give me a reason to quit, you know, your baby,,,, if I was pregnant it would be, like it would make me do it, it would be hard, but I would actually do it and I would for my kid” [31]
	“I didn’t want my kid, my son, to come out deformed or something, with something wrong with him” [39]
Pride in being a healthy mum	“need to be motivated to quit for yourself and supported by encouragement” [28]
	“I’m pretty proud of myself that I can just quit just like that. There’s a lot of people that can’t do that” [27]
	“I will do it when I am ready. When I want to do it so there is no pressure … I don’t want someone to tell me I have got to do it” [26]
	“If I could give up it would give me the world of good” [30]
	“for my health” [30]
Influence of women role models	“Mum’s voice was always in my head” [27]
	“I’m the doctor I know, so you have to do what I say and I’m sorry you don’t tell me what to do you are not my mother you know” [26]
	“I just said I’m trying to give up cigarettes and smoking and she’s like you know you can do it, you don’t need it and all of this sort of stuff. That gave me a bit of confidence in myself that I knew I could do it” [27]
	“mum used to rip me every time I used to smoke in front of her she said ‘you want to chuck them away I’d say yeah but I just kept smoking” [26]
Importance of family support	“the father tells me to give up” [30]
	“even though she smokes she doesn’t like me smoking” [30]
	“too hard when you have got like, when everyone in your house is smoking kind of thing, and you are the only one trying to quit” [31]
	“I just told everybody don’t give me smoke any day. And I kept on asking and they were like ‘no you told me not to give it to you’ and I am like ‘oh well” [31]
Understanding drivers for smoking	
Impact of stress and chaos	“All I know is that my smoking is aligned with times of chaos and stress” [27]
	“I knew it wasn’t good for me or baby at the time, but like it was just mores stress...because I had so much of it” [27]
	“She gave it away but then she could not deal with the stress that was going on” [26]
	“there’s heaps of stress in Aboriginal families. It doesn’t matter what it is, always stressing after something” [31]
	“I would say that it relaxes ya after you have a feed or a sit down, or a yarn with somebody, you are talking and you are smoking and yeah” [31]
Social acceptability of smoking	“smoking as a normal and accepted behaviour, a low health priority” [31]
	“Living with no smokers’ or at least that people around you don’t smoke around you. It’s pretty hard when other people are smoking around you” [28]
	“Well I am pregnant now and I smoke but it is just really hard to give up. I just can’t like everyone else around me just smokes and it is just hard I just can’t do it” [26]
	“Just people tend to say ‘oh it’s not going to hurt you know, it won’t affect the baby, it’s alright I did it when I was pregnant” [26]
Guilt and judgement	‘I didn’t want anyone to know that I’d started again, so I was hiding it for a bit” [27]
	“I made that choice, I wanted to smoke again so I did it. I’m glad this last time that I’ve quit for good” [27]
	“He has got breathing problems now and I reckon that’s smoking all the way through” [26]
Lack of information to support decision-making	“Would be more helpful for health professionals to “go through the pamphlets” [28]
	“More advertising in the cigarette pack itself for pregnant women, have a graphic picture” [28]
	“You have got to push more ‘this is what it is going to do to your child, your child could have this in so many years” [26]
	“The doctor should give us advice. He should know about the research and they should know the right information” [28]
	“They often talked to me about my smoking and they did say if you can slow down as much as you can, but try not to go cold turkey” [29]
	“They gave me patches and chew you try but like I didn’t feel comfortable taking them while I was pregnant” [29]
	“They just drum into you it’s more of a low body weight and that’s what starts them off wrong” [26]
Appreciating culturally responsive approaches	
Valuing indigenous programs	“midwife of the same culture … or a kaupapa Maori service, that is grounded in a Maori worldview and operated in accordance with Maori cultural protocols [quitline] couldn’t give me any information ..I’m trying to find a Maori midwife but its hopeless” [28]
	“Trying it in with the whole family more than the individual I think because then it’s not just one person going home … because I don’t think that works very well because it’s one person trying to preach” [26]
	“More readily available services for Maori women and easily accessible, ring and they come round and see me, especially if they are a Maori person” [28]
	“Most people listen to their elders. Like I can go to the doctor’s and listen to the midwives telling me not to do stuff, it’s like I don’t know you. Who are you? You’re just a doctor here that works here. You don’t know my life, you don’t know where I live, actually be like, I know what you’re going through, don’t worry, stop stressing Like they know more about that family and just be able to support more” [29]
Increasing accessibility of programs	“There’s more support needed or this one thing than there is anything else” [29]
	“Find out what the options are and allow her easy access to those options at no cost” [28]
	“It should be offered to every pregnant woman, that there are services available. Make it more aware that there are services out there, more than quitline, I didn’t know there are subsidised patches and gum” [28]
	“Doctors should prescribe things, I was quite shocked especially for a doctor not to” [28]
Needing something to replace smoking	“Just got to have something in my hands, It’s not that I like it” [30]
	“Would be helped to quit if they had something to occupy them, such as a programme that offered an “alternative”, that would keep them busy, “like a hobby, fitness”...interest or maybe a support group” [28]
	“No they’ve only offered me the gum once” [29]
	“I felt like having a cigarette this morning when I got up, but instead of doing that I walked the kids to school rather than driving them to school and you think oh that’s something I haven’t thought about trying maybe I’ll do that...I think hearing other people’s stories and how they cope with it is helpful” [29]

### Realising well-being and creating agency

#### Giving the best start to baby

Many women prioritised stopping smoking “so my baby would be healthy” once learning they were pregnant. The health of their baby was a paramount consideration despite experiencing difficulty stopping smoking. “I gave up smoking when I was about 7 months, it was hard for me but I just thought of my baby” [26]. Retrospectively, women who were not aware of the harms of smoking during pregnancy were grateful that they did stop.

#### Pride in being a healthy mum

Some women wanted to be smoke-free because this would mean that they themselves would be a healthier and therefore better mum; “I would have a lot more energy, and be able to do more things with my son and not be puffed” [27]. One women acknowledged that now as a mum she would be a role model for her child and “wouldn’t want them to think it is OK to smoke” [27]. Another mum felt a sense of needing to keep herself well in order to “see them grow up” [27]. Reducing the number of smokes or becoming smoke-free also instilled pride and confidence that they were making positive changes for themselves and baby.

#### Influence of female role models

The importance of other women to smoking cessation, particularly as positive role models “to support, praise, awhi (support) women” [28] was evident in the women’s experiences. This support gave women “confidence in myself, that I knew I could do it” [27]. The influence of indigenous women to stop smoking was valued higher than medical advice. “I have nothing but the utmost respect for my aunt whereas the doctor is just another Joe Blow” [29].

#### Importance of family support

Having encouragement and support from family and partners to stop smoking was a critical factor in women successfully becoming smoke-free during pregnancy. Being reminded to “think of the baby” [30], and positive messages encouraged self-belief. Some women told of how their partner trying to give up at the same time helped them to stay motivated and committed. In contrast to these experiences, other women expressed how hard it was when their “families won’t support you to quit because theytoo busy smoking themselves. They encourage your smoking even more” [31]. Some women’s families also directly disregarded their attempts to stop smoking by “saying, ‘no you’s can’t do it” [26] leading to self-doubt in their ability to stop.

### Understanding the drivers for smoking

#### Impact of stress and chaos

Many women identified competing issues that impeded their attempts to quit smoking while pregnant. Stress, difficult relationships and times of chaos within the women’s lives impacted on their capacity to prioritise smoking cessation. “I knew it wasn’t good for me and baby at the time, but like it was more stress … because I had so much of it, I just didn’t care what I was doing at the time” [27]. Smoking was also identified as self-medication to manage stress. One women described that smoking “stops me from stressing out, stops me from worrying about things” [30].

#### Social acceptability of smoking

Women spoke of their strong connection to their wider family and community and how within their indigenous community, smoking was acceptable and a lower health priority for them and their baby. Their role as a smoker gave them a sense of belonging and connection, they were concerned about social isolation if they stopped smoking.

#### Guilt and feeling judged

Guilt for some women “made me quit” [26], whereas others felt guilty that they “should be able to just stop for the health of my baby, but I just can’t” [26]. This led to women feeling ashamed and hiding that they smoked. Women felt guilt and regret that their smoking was the potential cause of their baby’s health problems. “my last one [pregnancy] was very traumatic just due to smoking, all my pregnancies were difficult due to my smoking” [31].

#### Inadequate information about smoking risks

Many women were uncertain of the risks to their baby during pregnancy and had received conflicting information. Women in many studies cited the biggest risk of smoking as “it makes baby small” [31], however some saw this as an advantage, particularly when they were warned about the risks of big babies in diabetic pregnancies, “oh I can’t quit because I am having a bigger baby or I want the baby to be small” [26]. In one Australian study, women reported not being told to quit smoking during pregnancy, but “only to reduce their intake” [29]. A common theme throughout studies was the belief cutting down the number they smoked or having breaks throughout pregnancy without smoking would be better for baby. Women reported receiving conflicting information about the risks of smoking cessation aids including nicotine replacement therapy and “the effect on baby” [28]. Many women reflected that if they had known earlier that they were doing harm to their baby, then they would have been more committed to stopping smoking “I had no signs of an unhealthy baby, so I continued to smoke” [27].

### Culturally responsive approaches

#### Valuing indigenous programs

Women reported current smoking cessation programs did not meet their values and preferences, recommending programs needed to be “more one on one” [29] with people who understood their background and “where the smoker is coming from” [28]. Women valued care from indigenous health workers and education and support involving “all the whānau (family)” [28]. Family-based care strengthened the impact of knowledge “the more we know, the more knowledge we have and we can share it with other people” [28]. Women recommended methods of delivery of education such as “yarning”, or visual, or “via music, listening to lyrics to do with stop smoking” [28].

#### Improving accessibility of programs

Smoking cessation programs were experienced as inaccessible and impersonal and failed to deliver care “I felt too disconnected from my support and they didn’t ring as promised” [28]. Some women described the benefit of out-reach services, “some people don’t have a phone and the fact that the service came to my home at a time convenient to me is a huge bonus, made it easier for me not to avoid” [28]. The lack of subsidisation and wide availability of nicotine replacement therapy prevented use [28]. Women suggested that there needed to be more advertising and campaigns about the risks of smoking during pregnancy and how to access support to quit.

#### Needing something to replace smoking

Some women wanted there to be more to smoking programs than just smoking cessation advice, and include support for their overall well-being. Women identified smoking as an activity that built social connections, which highlighted the opportunity to use other joint activities to build and maintain social relationships; “I reckon if we had something to do, you know like keep us busy, take our mind off smoking, like arts and craft” [31]. Women found hearing advice from others on ways “they cope with it is helpful” [29]. Women experienced being more isolated and less distracted when pregnant. This gave more time to think about smoking and made it harder to stop; “if you do other stuff, you’re not going to worry about it, so that’s how you cut down” [31].

## Discussion

This systematic review has synthesised perspectives, attitudes, expectations, and experiences of indigenous women from qualitative research investigating smoking cessation in pregnancy. We found only seven eligible studies involving 250 indigenous women from Australia and New Zealand. We had also searched for studies from Canada, Taiwan and the United States - developed countries where the indigenous peoples have been colonised and where we might have expected to see some common experience with widespread social and socio-economic marginalisation, and gross health inequities [32]. Despite those similarities there are vast differences in delivery of health services between and within these countries, ranging from mainstream services who are generally unable to meet indigenous need to indigenous health care systems that are under-resourced and struggle to cope with demand [33]. The delivery of smoking cessation programs is similarly diverse.

We identified three central themes across these services. First, realising well-being and creating agency were expressed as giving the best start to baby, pride in being a healthy mum, and the importance of female role models and family support. Second, understanding the drivers for smoking included the impact of the stress and chaos indigenous women often experience in their lives, the relative social acceptability of smoking within indigenous communities, the pervasive forces of guilt and judgment and the lack of information and knowledge to support decision-making. Finally, the women identified their appreciation for culturally responsive approaches to cessation valuing programs designed specifically for and by indigenous people that were easily accessible and that acknowledged the need for programs to provide an alternative to smoking.

These findings broadly reflect those found in a systematic review of Aboriginal and Torres Strait Islander women [34] in which social norms and stressors within the Aboriginal community perpetuated tobacco use and there was insufficient knowledge provided to smokers in ways that supported smoking cessation. Another systematic review also indicated that community-based knowledge of the harmful effects of smoking did not translate into smoking cessation [35]. The findings in the present systematic review suggest that this knowledge might be effectively translated by building on the self-agency and determination of woman to have a healthy baby, be healthy mums and incorporating the views of influential female peers into programs. Our review indicates that current smoking cessations programs need to be closer aligned to the values of indigenous women and communities.

Other studies have reported the challenges faced by health professionals to delivering smoking cessation advice and care during pregnancy [36, 37]. Lack of support from health professionals has previously been reported as a common barrier to smoking cessation during pregnancy and for indigenous peoples, the pervasive effect of cultural and historical norms [38].

Women in these studies experienced receiving inconsistent information and education from health care professionals and others in their community, as has been previously described [35]. This inconsistency in information caused confusion and doubt about the potential harms of smoking, especially if women had known of other “healthy” babies whose mothers had smoked. Women also recommended programs would be more successful if they helped them to address some of the wider issues that led to their smoking and also include other activities that replaced smoking and strengthened a sense of community. Acknowledging the absence of evidence to support smoking cessation in pregnant Aboriginal and Torres Strait Islander women, Gould [40] developed a pragmatic guidance for health practitioners that includes: screening the patient, counselling, initiation of NRT, making a personal Quit Plan, follow-up support, and utilising ancillary resources. Although this guide may respond to some needs, we recommend a more culturally responsive approach that includes collaboration and partnership with indigenous people built on cultural values. This approach has been successful in New Zealand in the Safe Sleep SUDI prevention programme designed around Safe Sleep devices that were able to be deployed in the shared bed to prevent sudden infant death [41, 42]. This program utilised traditional crafts (weaving with flax) to support a traditional value (bed-sharing) that was widely supported by Māori women elders in the creation of this safe infant sleep space and thus addressing cultural needs and increasing support available to new mothers. The findings of this review suggest that this kind of cultural resonance and support is needed for smoking cessation during pregnancy developing a culturally meaningful and accessible programme that provides support and is empowering of pregnant indigenous women.

Understanding successful interventions and linking these to the results of this review will help to build stronger smoking cessation programs that specifically target indigenous women. Future research should include the development of smoking cessation programs built on successful interventions that align with the findings of this review.

## Conclusions

Future interventions and smoking cessation programmes might be more effective and acceptable to indigenous women and families when they harness self-agency and the desire for a healthy baby, recognise the high value of culturally responsive approaches and indigenous peer involvement and embed a social focus in place of smoking as a way to maintain community support and relationships. Development and evaluation of smoking cessation programs for pregnant indigenous women and families is warranted.
